# A Global Comparison of the Human and *T. brucei* Degradomes Gives Insights about Possible Parasite Drug Targets

**DOI:** 10.1371/journal.pntd.0001942

**Published:** 2012-12-06

**Authors:** Susan T. Mashiyama, Kyriacos Koupparis, Conor R. Caffrey, James H. McKerrow, Patricia C. Babbitt

**Affiliations:** 1 Department of Bioengineering and Therapeutic Sciences, California Institute for Quantitative Biomedical Research (QB3), University of California San Francisco, San Francisco, California, United States of America; 2 Center for Discovery and Innovation in Parasitic Diseases, and Department of Pathology, QB3, University of California San Francisco, San Francisco, California, United States of America; McGill University, Canada

## Abstract

We performed a genome-level computational study of sequence and structure similarity, the latter using crystal structures and models, of the proteases of *Homo sapiens* and the human parasite *Trypanosoma brucei*. Using sequence and structure similarity networks to summarize the results, we constructed global views that show visually the relative abundance and variety of proteases in the degradome landscapes of these two species, and provide insights into evolutionary relationships between proteases. The results also indicate how broadly these sequence sets are covered by three-dimensional structures. These views facilitate cross-species comparisons and offer clues for drug design from knowledge about the sequences and structures of potential drug targets and their homologs. Two protease groups (“M32” and “C51”) that are very different in sequence from human proteases are examined in structural detail, illustrating the application of this global approach in mining new pathogen genomes for potential drug targets. Based on our analyses, a human ACE2 inhibitor was selected for experimental testing on one of these parasite proteases, TbM32, and was shown to inhibit it. These sequence and structure data, along with interactive versions of the protein similarity networks generated in this study, are available at http://babbittlab.ucsf.edu/resources.html.

## Author Summary

Human African trypanosomiasis (HAT) is caused by the protozoan parasite *Trypanosoma brucei*. HAT is fatal unless treated, yet the current treatment itself can cause death. New treatments are urgently needed. Our study focuses on proteases, which are enzymes that break down proteins. Because of their roles in many centrally important biological processes, proteases are targets for drugs to treat a variety of diseases including parasite infection. The recent explosion of protein sequence and structure information in public databases has made surveys of proteins on a genomic scale possible. However, collecting specific data of interest from diverse databases and synthesizing them in a way that is easy to interpret can be difficult. We used *T. brucei* and human protease sequences, crystal structures, and models to create network views that show how proteases cluster by similarity. Such views are valuable not only for understanding the evolution of the protein repertoire in each species, but also can give important clues for drug design. Two *T. brucei* protease groups (“M32” and “C51”) that are very different in sequence from human proteases were examined in structural detail. Based on our analyses, a human ACE2 inhibitor was selected for experimental testing on one of these parasite proteases, TbM32, and was shown to inhibit it.

## Introduction

The recent explosion of sequence and structure information in the public databases has made possible surveys of proteins on a genomic scale. Structural genomics initiatives have rapidly increased our knowledge of protein structures, and this has provided a foundation for better elucidating reaction mechanisms and ligand binding, for understanding substrate specificity, and for creating many new three-dimensional (3D) structural models [1]. One use of such information is to inform drug discovery. For example, Vidovic *et al* examined human protein-tyrosine phosphatases as targets for rational drug design [2]. Rational drug design has been shown to produce selective drugs: for example, a number of effective cancer drugs have been produced that have fewer side effects than traditional, cytotoxic drugs [3]. Evaluating potential off-target effects is an important consideration in the process, and surveying homologs of target proteins can reveal unanticipated interactions [4], [5]. Conversely, some drugs show efficacy with unanticipated targets making them useful in treating diseases other than those for which they were designed. For example, Kinnings *et al.* used a computational approach to compare proteins with similar binding sites to those of the targets of commercially available drugs and found that a drug approved for Parkinson's disease may be effective for treating tuberculosis [6]. Thus, the larger context provided by examining the differences between structurally related proteins may aid in the design of more selective drugs, while study of their similarities can give clues for new starting points in drug design.

While the public databases are rich with sequence and structure data, retrieving specific data and synthesizing the information into views that are intuitively interpretable is not a trivial task, even for an experienced user of bioinformatics tools. The central idea behind our study is to take the approach illustrated by Vidovic *et al* one step further and construct genome-wide views for more than one organism; specifically, for a host and parasite, allowing cross-species comparisons. We constructed these views using sequences and structures of the proteases of the pathogen *Trypanosoma brucei* and its human host *Homo sapiens*. The full diversity of a set of sequences or structures is often termed “sequence space” or “structure space.” To visualize the information, we used similarity networks, whereby sequences or structures are clustered graphically by similarity. Such networks represent a powerful way to visualize relationships across large sets of sequences and structures [7]. To construct the structure similarity networks, existing crystal structures and homology models, as well as newly created models, were utilized.

Proteases were chosen for this computational study because a number of these proteins have been validated as druggable targets and many have available structures. Protease inhibitors are currently under investigation to treat various parasite infections, cancer, HIV, hypertension, and diabetes [8]–[18]. Here, we employ the nomenclature of the protease database MEROPS [19] in labeling proteases by the evolutionary units of family and clan (see Methods). Proteases catalyze the hydrolytic breakdown or processing of proteins and account for about 2% of all expressed genes [20]. The set of an organism's proteases expressed at a particular time or circumstance has been called its “degradome” [21]. Here, we use the term to refer more generally to all the active proteases coded by an organism's genome.

The parasite degradome targeted in this study is that of the protist *T. brucei*, which causes human African trypanosomiasis (“HAT”) or sleeping sickness, a disease that affects an estimated 50,000 to 70,000 people, mostly in sub-Saharan Africa [22]. HAT is one of a number of ‘neglected’ tropical diseases that primarily afflict the poor [15], [22]. The few existing treatments for such diseases often have severe side effects. Without drug treatment, HAT is often fatal; yet the standard drug used to treat infection of the central nervous system is itself often lethal [23]. *T. brucei* is related to two other human pathogens, *Trypanosoma cruzi and Leishmania major*, that share many physical characteristics [24]. These three species are referred to as the “Tritryps” [24]. As will be illustrated here for *T. brucei,* knowledge about well-characterized proteins in the other Tritryp species is valuable for inferring characteristics about related but less well-characterized proteins in the target species, for example, by enabling the creation of homology models.

The objectives of this study were first to compare the array of proteases in the human host with that of the *T. brucei* pathogen and to determine the breadth of sequence space in each organism that was covered by 3D structure. Secondly, we aimed to use the similarities and differences within and between protease sequence and structure similarity groups to obtain insights into possible new targets for drug design. The global views produced here will also be useful for guiding phylogenetic and other more detailed studies comparing proteases of the parasite and its human host. We found that structure coverage of sequence space in human and parasite is broad, making global structural comparisons both feasible and informative. To illustrate how these results may be used to better understand structurally related human and parasite proteases, we include a detailed structural evaluation of two groups of parasite proteases that may have potential as new drug targets. For one of these protease targets, TbM32, we predicted and experimentally confirmed its inhibition by a known human drug.

## Methods

We used a protease reference dataset (“pep82”) composed of peptidase domain sequences from the manually curated database MEROPS release 8.2 downloaded from http://merops.sanger.ac.uk [19]. The dataset pep82 includes only the regions of sequences that correspond to peptidase-like domains, rather than the full-length sequences that are also available from MEROPS. Families in MEROPS are defined by sequence similarity where each family member has statistically significantly similarity to at least one other family member in at least the region of the sequence where the catalytic residues are located [25]. The first letter of a family name designates the protease catalytic type defined by the characteristic moiety needed for catalysis: A (aspartic (catalytic type); aspartic acid (characteristic moiety)), C (cysteine), G (glutamic; glutamic acid), M (metallo; metal ligand), S (serine), and T (threonine). For example, “S01” is a prominent family of the serine protease catalytic type. MEROPS also categorizes proteases into more remotely related groups called clans that include distant homologs identified using structure-based comparisons. Clans are denoted by two letter labels where the first letter represents the family's catalytic type and the second differentiates clans. A first letter of “P” indicates that a clan contains families of one or more of the serine, threonine, and cysteine catalytic types. A dash instead of a second letter indicates that a family is not yet formally assigned to a clan. While families and clans indicate groupings that are likely to be related evolutionarily, the catalytic type defines groups only by whether members have shared catalytic moieties; thus some members of a catalytic type may not be related.

### Identification of *T. brucei* proteases predicted to be active

Predicted *T. brucei* proteins were from the *T. brucei* genome project (9,192 sequences, file “Tb927_Proteins_May08_v4.fas” downloaded from ftp.sanger.ac.uk) [26], and will be referred to hereafter as “Tb_proteins.” To identify protease-like sequences, we used a protocol similar to that used by Berriman *et al.* [27]. The Tb_proteins were BLAST searched using blastp [28] against pep82, and the results limited to those with *E-*value cutoff ≤1e-4, which yielded 477 “provisional proteases.” Note: scientific “e notation” is used to express *E*-values, e.g., where 1e-4 represents 1×10^−4^. Because this *E-*value is rather non-stringent, these hits were compared with similar sequences in Swiss-Prot (downloaded November 2, 2008), a manually curated set of protein sequences known to have reliable annotations [29], [30]. Additionally, the provisional proteases were searched against 219 profile hidden Markov models (profile HMMs) from Pfam [31] version 22.0 that corresponded to MEROPS peptidase families (*personal communication with Neil Rawlings*) using the program HMMER (v2.3.2; trusted cutoff) [32]. Because profile HMMs define the likelihood of finding particular amino acids in a column in a multiple sequence alignment of relevant sequences, they are helpful in identifying distantly related proteins by scoring more highly the presence of specific regions and residues important to a known family. [32]. *T. brucei* sequences were removed as false hits if they were similar to SwissProt sequences annotated with non-protease functions or if they matched a Pfam model to non-protease families. After removing false hits, 251 “putative proteases” remained.

Predicted active proteases were identified using tools at the MEROPS website where metal-binding and active site residue (MASR) sites for a sequence can be predicted based on BLAST alignments to pre-computed alignments of families. Of 251 putative proteases, 127 were predicted to be active proteases and trimmed to remove non-peptidase regions by examining the results of our original BLAST searches of *T. brucei* sequences against pep82 and removing regions that were not included in the alignments to the MEROPS peptidase domains. This final set of predicted active protease sequences is called “Tbpep.”

### Identification of predicted active human proteases

Unlike *T. brucei,* human sequences were already well-curated in MEROPS with MASR data readily available for each sequence. Of the 958 human peptidase domain sequences from pep82, 574 were predicted to be active according to the MASR data; this set is hereafter called “Hspep82.” Twenty additional human peptidase sequences were identified from the Mammalian Degradome Database (“MDD”), a manually curated dataset of proteases from the Lopez-Otin group (http://degradome.uniovi.es) [33] that were found to be dissimilar to Hspep82 sequences but predicted to be active peptidases (by BLAST searches against pep82 and MASR prediction similar to the procedure for *T. brucei*). These 20 sequences were trimmed to remove non-peptidase regions and added to the 574 sequences above. This final set of 594 predicted active human peptidase sequences is called the “HsLOpep82” dataset.

### Identification of crystal structures corresponding to peptidase sequences

PDB entries representing Tbpep and HsLOpep82 sequences were found by BLAST searches of the PDB protein sequences and those with good resolution (≤3.5 Å) were kept for analysis. The 150 human and one *T. brucei* pdb files were trimmed to remove non-peptidase regions. This was done because a trial test showed that structure similarity detection between peptidases can be obscured if non-peptidase regions are included in structures being compared (*data not shown*).

### ModBase models for peptidase sequences

For sequences without crystal structures, models were taken from ModBase (http://modbase.compbio.ucsf.edu) [34], a large database of comparative structural models (homology models) created using the Modeller program [35]. Only good quality models, as determined by using the recommended cutoff of ModPipe Protein Quality Score (MPQS) >1.1, were used in this analysis. There were 174 human models and 48 *T. brucei* models initially identified. Models were checked to make sure that they spanned the peptidase regions of the target sequences, and those with unacceptably short or incorrect regions were discarded. 141 human and 47 *T. brucei* models passed this check. These were then trimmed to exclude non-protease regions.

### ModWeb models for peptidase sequences

Because modeling is computationally- and time-intensive, we created new models only for representatives of clusters of similar sequences for which no structure (crystal structure or ModBase model) existed. Representative sequences of clusters with ≤40% sequence identity (“sequence ID,” clustered with CD-HIT [36]) that had no structures were submitted to ModWeb, a homology modeling web server that utilizes Modeller (http://modbase.compbio.ucsf.edu) [34]. This yielded 51 and 23 new human and *T. brucei* models with MPQS >1.1, respectively.

### A note on model quality assessment

It has been shown that better quality models (about 1.5 Å or better root mean square (RMS) error between template and model) generally result from using templates with ≥30% sequence ID to the target sequence [37]. However, sequence identity alone can be misleading. Additional factors can be important indications of model quality such as how much of the template sequence aligns well to the target and whether inter-atomic distances in the model are similar to those seen in real proteins. The MPQS reported in Modeller is a composite score that includes a number of such factors. Of the models included here, 80% have ≥30% sequence ID to their templates.

### Additional modeling

An updated model for the *T. brucei* M32 sequence (TbM32) was created using the program Prime (Prime 2.0208, Maestro 8.5207, Schrodinger LLC, Portland, OR). The structure for a *T. cruzi* M32 protease (PDB code 3DWC) [38] was used as the template for modeling because it possesses a higher sequence ID to TbM32 than the 1KA2 structure previously used for the ModBase model (72% vs. 33%).

### Creation of sequence similarity networks

**Figure 1:**
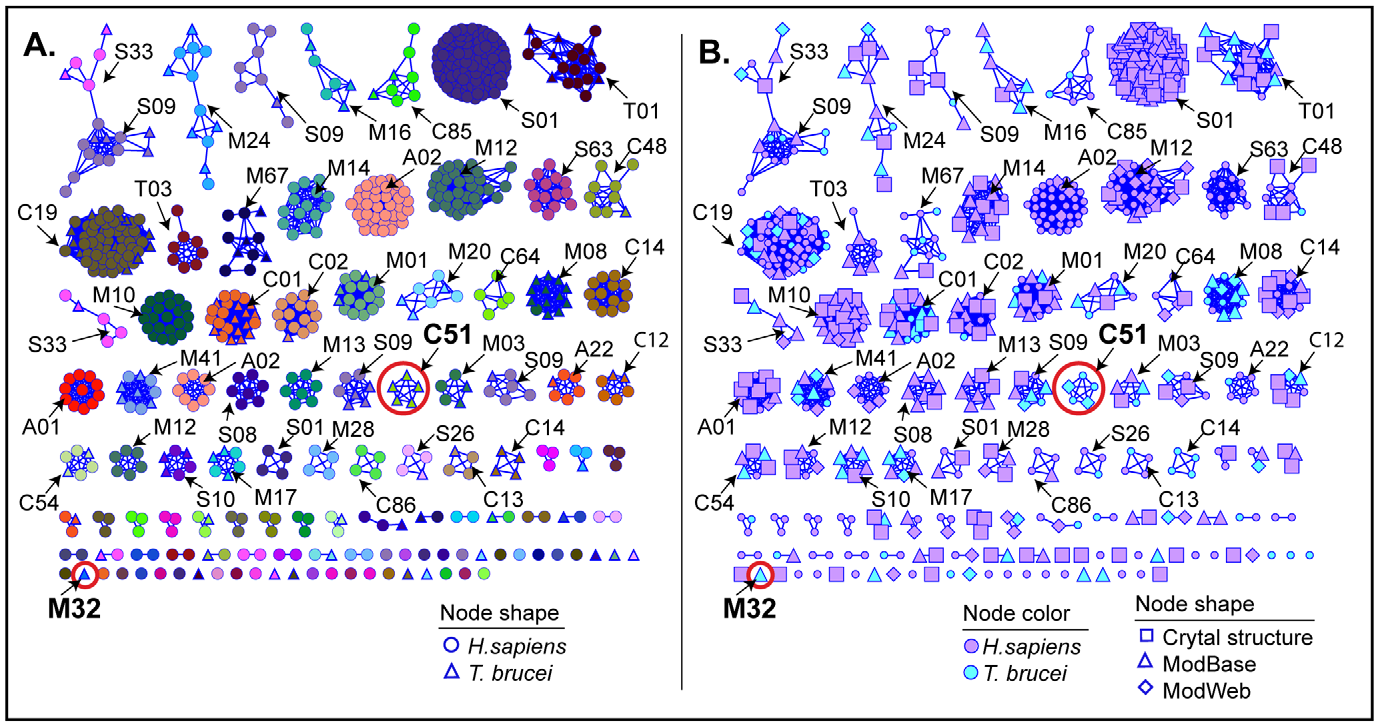
Global view of predicted active proteases of human and *T. brucei* showing sequence similarity relationships. Protease sequences are represented as nodes, and similarity relationships between sequences better than the threshold (BLAST *E-*value ≤1e-5) are depicted as “edges” or lines between nodes. In the network are represented 594 human and 127 *T. brucei* sequences (total of 721 nodes and 10,188 edges). (A) Distribution by family of proteases. Nodes for human sequences are represented as circles and for *T. brucei* sequences as triangles, and are colored by MEROPS-associated family (see Methods). Families of some of the larger clusters are labeled, and the parasite-specific C51 and M32 clusters are circled in red. (B) Structure coverage of sequence space is broad in human and *T. brucei*. The same sequence similarity network as in panel A is shown except that it is color-coded by species and nodes are enlarged and designated by different shapes to denote if a crystal structure or model exists for that sequence. Node shapes: square = crystal structure; triangle = ModBase model; diamond = ModWeb model; small circle = no structure.

All-by-all blastp scores were computed on the 594 HsLOpep82 and 127 Tbpep sequences (total of 721 nodes), and the data viewed with the network visualization program Cytoscape [39] using the “organic” layout setting, which clusters nodes more tightly if they are more highly connected; a BLAST *E-*value threshold of ≤1e-5 was required for drawing edges between any two nodes, resulting in 10,188 edges (Figure 1). This corresponds to ≥40% sequence ID for alignments ≥50 residues. Nodes are color- and shape-coded by species, MEROPS family (according to the family of the best BLAST hit to pep82 sequences as described above), and structure representation.

### Creation of structure similarity networks

An all-by-all pairwise structure similarity comparison was performed using the program FAST [40] on the trimmed pdb files (crystal structures, ModBase models, and ModWeb models) for the 71 *T. brucei* and 342 human predicted active peptidases (total 413) with structure representation. The data were visualized with Cytoscape (“organic” layout; a threshold of normalized FAST score (SN) ≥4.5 was used to draw edges. This score is well above the minimum cutoff (1.5) stated by the authors of FAST to be statistically significant. Nodes were color- and shape-coded by species, MEROPS family, and structure representation.

### Structure and sequence alignment and visualization

Protein structures were aligned, visualized, and root-mean-square deviation (RMSD) values calculated using the MatchMaker tool from the Chimera software package [41]. Default settings were used, with aligned pairs of atoms ≤2 Å included in the RMSD calculation. Alignments with similar RMSDs but with more aligned pairs within this threshold indicate higher overall structural similarity. Multiple sequence alignments (MSAs) were created using MUSCLE [42] and visualized with the program GCG SeqLab (Wisconsin Package Version 10.3, Accelyris Inc., San Diego, CA). For the *T. brucei* protease of family M32 (TbM32) and human angiotensin-converting enzyme 2 (ACE2, PDB code 1R4L), the structure-based sequence alignment was visualized with ESPript [43] (http://espript.ibcp.fr/ESPript/cgi-bin/ESPript.cgi).

### Structure similarity searching via PDBeFold

To find structural relatives for the *T. brucei* C51 family in the PDB, the structure alignment server PDBeFold [44] at ebi (www.ebi.ac.uk/msd-srv/ssm) was utilized by submitting the ModWeb model for the amidase domain of trypanothione synthetase-amidase (“TbTSAAm”) and specifying 40% as the lowest acceptable match of secondary structure elements for both query and target.

### Expression and purification of polyHis-tagged recombinant TbM32

The TbM32 gene was amplified by PCR from genomic DNA with Phusion polymerase using sense (5′ - GCGCGC**CATATG**ATGAAGGCATACAAAGAGCT - 3′) and antisense (5′ - ATGCAT**GTCGAC**TCAGTTGGCATCGTCACGGT -3′) primers. The PCR product was cloned into the pET28a expression vector (Invitrogen, Carlsbad, CA) and the N-terminal polyhistidine-M32 expressed in *E. coli* strain BL21-DE3. The recombinant enzyme was purified in two steps: first using a Ni-NTA slurry, then further purified on a Ni column (5 mL HisTRAP FF column) using an ÄKTA FPLC system (GE Healthcare Life Sciences, Piscataway, NJ) at 4°C from which the protein was eluted using a 0–250 mM imidazole linear gradient in 2 column volumes. Active fractions were analyzed by gel electrophoresis, and pure samples were combined, flash frozen and stored at −80°C for future use.

### Enzyme assays

Recombinant TbM32 (2 µM) was assayed using the synthetic carboxypeptidase substrate FA {N-(3-[2-furyl]acryloyl)}-Phe-Phe (“FAFF”, BACHEM, Torrance, CA) as substrate (100 µM) in 50 mM Tris/HCl, pH 7.4. Initial steady-state velocity (“Vi”) was determined by continuous assay for a range of substrate concentrations at 340 nm with a SpectraMax Plus platereader (Molecular Devices, Sunnyvale, CA). Vi was calculated as milliunits/min using SoftMaxPro software (Molecular Devices). For inhibition studies, protease and inhibitor were pre-incubated for 30 min at room temperature prior to adding substrate. Concentrations of inhibitors and controls were: 10 µM of the ACE2 inhibitor 28FII (3-{[1-(2-acetylamino-3-methyl-butyryl)-pyrrolidin-2-yl]-hydroxy-phosphinoyl}-2-benzyl-propionic acid, active diastereoisomer, a gift from the laboratory of Vincent Dive [45]), 10 µM lisinopril (ACE inhibitor; Toronto Research Chemicals, North York, Ontario, Canada), 100 µM 1,10P (1,10 phenanthroline, a divalent metal chelator, as a positive control; Sigma-Aldrich), and 1% DMSO (negative control; Sigma-Aldrich, St. Louis, MO). Measurements were taken in triplicate and statistics were calculated using the software R v2.9.2 [46] by running ANOVA and a TukeyHSD post-hoc test.

## Results and Discussion

### Overview

**Figure 2:**
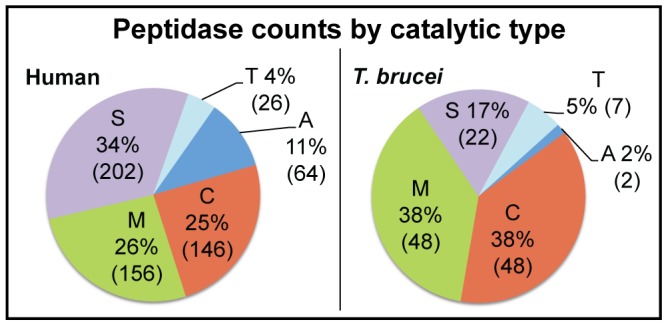
Distribution by catalytic type of peptidases predicted to be active in humans and *T. brucei*. In humans, proteases of catalytic type S (where the catalytic moiety is serine) is dominant, but metallo (type M) and cysteine (type C) peptidases are also abundant. In contrast, in *T. brucei,* serine peptidases are less abundant, and cysteine and metallo proteases are equally prominent. Other main catalytic types in each organism include the threonine (type T) and aspartatic (type A) proteases. Catalytic types were assigned by catalytic type designated in the family of the closest BLAST hits to MEROPS sequences.

**Figure 3:**
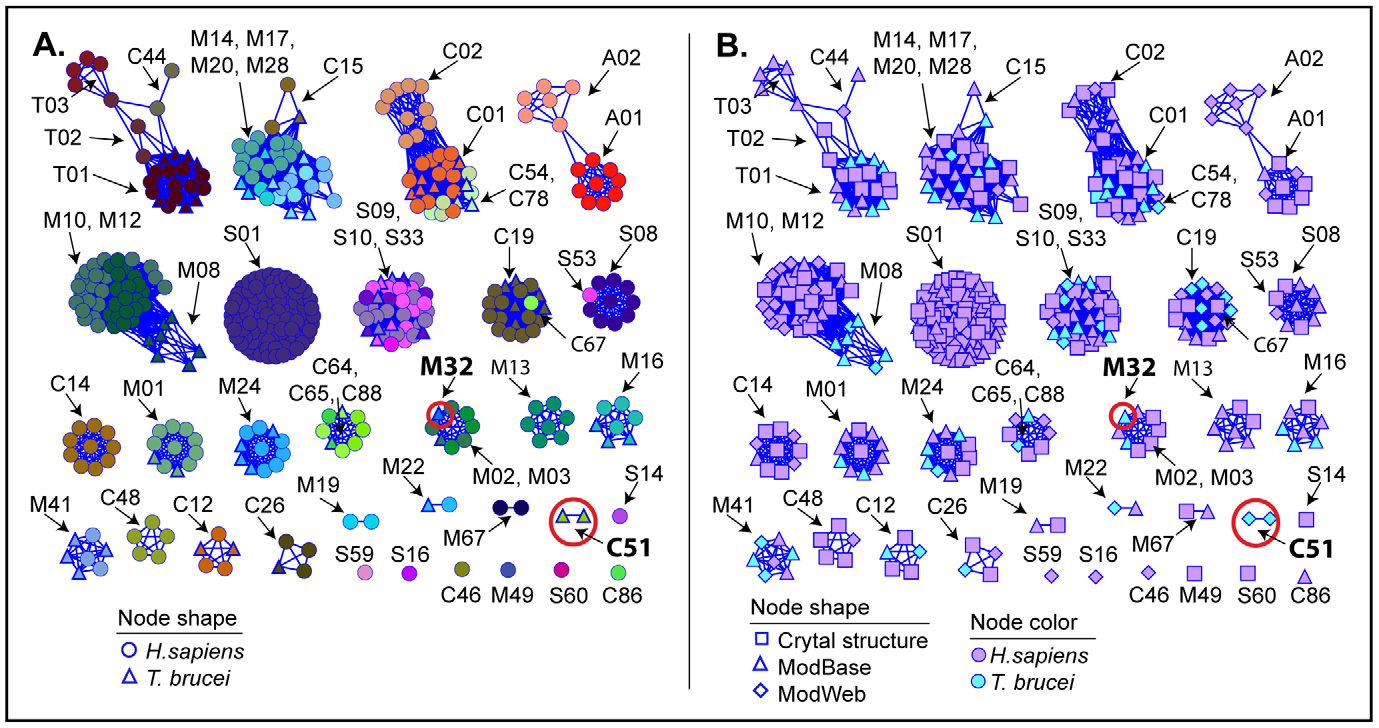
Structure similarity network of human and *T. brucei* proteases using crystal structures and models. Nodes represent experimentally characterized (crystal structure) or modeled structures and edges represent pairwise structural similarity above the structural similarity threshold (FAST SN score ≥4.5). Nodes for 342 human and 71 *T. brucei* are shown in the network (total of 413 nodes and 7,234 edges). The two *T. brucei-*specific families (TbM32 and C51) highlighted in the sequence similarity network shown in Figure 1 are circled in red. (A) Nodes are colored by MEROPS-associated family, revealing cross-family structural relationships. Human structures are represented as circles and *T. brucei* as triangles. (B) The same structure similarity network as in panel A is painted by species and structure representation. Nodes are color-coded by species and node shape corresponds to type of structure representation for that sequence: square = crystal structure; triangle = ModBase model; diamond = ModWeb model. In contrast to *T. brucei,* there are a large number of experimentally characterized (crystal) structures for humans, but many *T. brucei* structures can be modeled.

The sequence similarity network (Figure 1A) reveals on a global scale the diversity of families of proteases predicted to be active in humans and *T. brucei*, showing the protease families that are most prevalent in both organisms as well as those that reflect the greatest differences between them. Figure 2 shows the overall distribution of peptidases by catalytic type. The network shown in Figure 1B shows that when models generated by comparative structural modeling (homology models) are included along with crystal structures, most of *T. brucei* and human sequence space is well covered by three-dimensional structures (See Methods and the note on homology modeling below for a discussion of model quality). This made it feasible to create the global structure similarity network (Figure 3) that clusters human and parasite proteases by similarity of 3D structures. In similarity networks, large numbers of proteins can be viewed in a visually meaningful way. The proteins are represented as nodes (points) and similarity scores above a statistical significance threshold cutoff are expressed as edges (lines) drawn between the nodes. The greater the number of interconnections among the proteins within a grouping, the closer they are drawn together. It should be noted that the placement of such clusters as they are laid out in network views such as shown in Figures 1 and 3 is done roughly by size so proximity between separate clusters does not imply relatedness.

In the sections that follow, the overall patterns that emerge from the degradome landscapes in these network views are discussed, along with new hypotheses about parasite and host biology based on these comparisons. We also include a detailed structural examination of two protease groups, M32 and C51, that are very different in sequence from human proteases and may have potential as drug targets.

### The sequence similarity network provides a visual catalog of human and parasite proteases

In the sequence similarity network in Figure 1A, nodes are color coded by assigned MEROPS protease family (see Methods for definitions of family, clan, and catalytic type). There are five times as many human proteases (594 sequences) as *T. brucei* proteases (127), representing 71 and 37 different families, respectively. It has also been observed that in the degradome of the parasite *Schistosoma mansoni,* the parasite has fewer proteases representing fewer families than humans [27], but little work has been done to address in detail why this may be a general trend, though this may involve the specialization of parasites. In general, serine, cysteine, and metallo catalytic type proteases dominate both degradome landscapes. There are no glutamic proteases predicted to be active in humans, and this is also the case in *T. brucei.* Half (35) of the total families (73) in the network have both parasite and human members. However, there are a large number of families in humans (36) that are missing in *T. brucei*, and two families (C51 and M32) are specific to the parasite. Figure 2 shows the distribution of human and *T. brucei* peptidases by catalytic type, and Supplementary Table S1 shows the counts by family and the more remotely-related grouping of clan.

Serine proteases comprise the most abundant catalytic type of proteases in humans with 202 members. Among all species, the serine protease catalytic type is known to be a large category of proteins containing a number of independently-evolved families from different clans representing a wide variety of functions [47]. In humans, the largest family in this catalytic type is the S01 (trypsin and chymotrypsin) family (115 members), with members that have well-known roles in digestion as well as in blood coagulation and immunity [47]. S01 is also the largest family by far of any catalytic type in humans, with the second largest family (C19) having 51 members.

In contrast to humans, cysteine proteases (48) predominate over the serine protease catalytic type (22) in the *T. brucei* degradome (Figures 1A and 2). Cysteine proteases have functions in virulence, immunoevasion, and enzyme activation in parasites and are the subject of active research [48], [49]. There are 146 cysteine proteases in humans predicted to be active. Before the Tritryps genomes were available, previous work indicated that the majority of all proteases detected in the Tritryps were cysteine proteases [48]. However, our network using genomic data shows that metalloproteases are just as numerous as cysteine proteases both in *T. brucei* (48) and in humans (146) suggesting this may be a rich area for future studies.

Notably, the S01 family is devoid of *T. brucei* sequences (of several S01 homologs in *T. brucei,* all are predicted to be inactive). Although known to be generally quite distinct structurally, cysteine and serine proteases have mechanistic similarities due to the chemical relatedness of the active site sulfur and oxygen of cysteine and serine, respectively [49]. Both act as nucleophiles on the peptide bond, but sulfur is the better nucleophile. It may be that some ancestral C01 members were superseded by serine proteases that were somehow functionally superior for humans. Given the importance of the S01 proteases in human biology [47], [50], the absence of active members of this family in the parasite underscores significant differences with parasite biology, as has been noted previously [48], [49].

The sequence similarity networks in Figure 1 use only sequence data from human and *T. brucei* to create the groupings shown resulting in some differences from assigned MEROPS family classifications which assigns some divergent proteases to the same family based on sequences from all known proteases from all species. For example, Figure 1 shows that some families (e.g., family S01) are composed of more than one cluster, revealing great diversity within these clusters that may be interconnected if sequences from other species are included. More divergent structure relationships are evident in the structure similarity network discussed later.

### Structural coverage for the human and parasite degradomes

Figure 1B shows the same sequence similarity network as in Figure 1A, but here the nodes are color-coded by species, and sequences with structure representations (crystal structures or homology models) are denoted with larger nodes. The human degradome is covered much better in terms of crystal structure than that of *T. brucei:* when the networks were initially constructed, there were 150 crystal structures for human proteases, but only a single crystal structure for *T. brucei*, the cathepsin L-like cysteine protease rhodesain (from *T. b. rhodesiense*) [51]. When good quality models are considered, coverage of the *T. brucei* degradome becomes comparable to that of humans. In Figure 1B, 61% of the clusters with *T. brucei* members have structure representation and 67% of clusters with human members also have structure representation. The inclusion of homology models in the network increases overall structure coverage by about 50%.

There are 8 sequence similarity clusters with three or more members that have no structure representation (Supplementary Table S2) and so may be good targets for structural characterization. The largest of these clusters are C85 (6 human, 2 *T. brucei* sequences) and A22 (5, 1).

### A note on homology modeling

Although models are not generally considered to have the same accuracy as crystal structures, they can give valuable structural information. It has been shown that a good quality homology model generally results from using a template (crystal structure used to guide the modeling) with ≥30% sequence ID to the target sequence (protein to be modeled) [37]. At this level, there is expected to be about 1.5 Å or better root mean square deviation (RMSD) between the model and the actual structure, and the fold and many of the details are likely to be accurate. Only good quality models were included in our analyses (see Methods for details). We compared the model for a *T. brucei* cathepsin B-like protease in the network (TbCatB) with the crystal structure (PDB 3HHI [52]) that was solved for this protease after the networks were constructed, and found that the model was quite accurate. The overall structures aligned well (RMSD = 0.67 Å over 214 atom pairs) and the active site residues also aligned closely (RMSD = 0.51 Å over 33 atom pairs). The network model for TbCatB was based on human procathepsin B (PDB 3PBH; 48% sequence ID to the target). For comparison, two structures of the same protein, human ACE, solved by the same lab but bound to different inhibitors (1UZE and 1UZF bound to enalaprilat and captopril, respectively) have an RMSD of 0.22 Å over 574 atom pairs.

### The structure similarity network shows three-dimensional (3D) structural similarity relationships of proteases that may be distantly related

**Figure 4:**
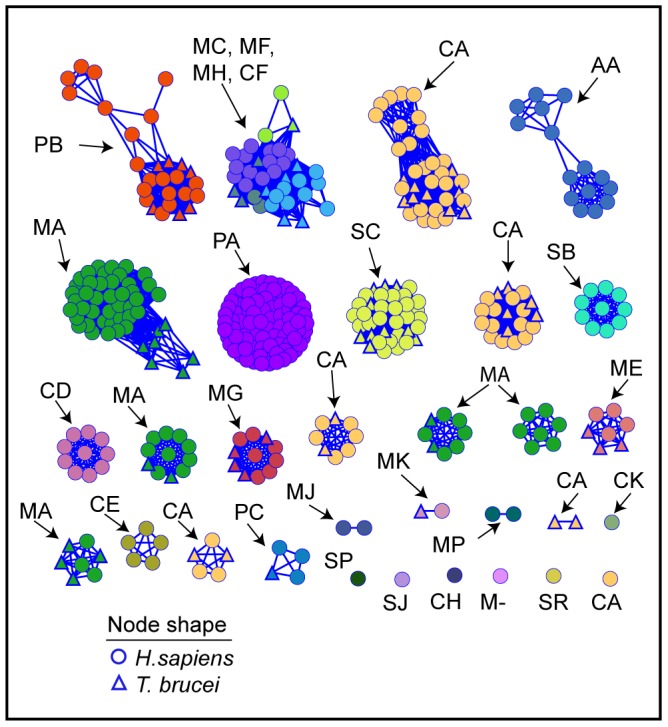
Structural similarity network of human and *T. brucei* proteases labeled by clan. The same network as in Figure 3 is colored here by assigned MEROPS clan (see Methods). One cluster is composed of multiple clans (MC, MF, MH, and CF).

Figure 3 shows the structure similarity network in which human and *T. brucei* crystal structures and homology models are clustered by 3D structure similarity. Figure 3A is colored by MEROPS family, and 3B by structure (crystal structure or model). There are fewer clusters than in the sequence similarity network, not only because some sequences lack structure representation, but also because some divergent sequences that are not connected in the sequence similarity network share similar structures (and are connected with edges in the structure similarity network). This reflects the phenomenon that protein structure evolves more slowly than sequence [53]–[55]. Figure 3A shows that a number of structure similarity clusters have members from more than one MEROPs family, underscoring the sequence divergence within clusters. Structure similarity is often used as evidence, along with functional similarity, that proteins with divergent sequences are evolutionarily related (i.e., are homologs) [56]–[58]. Most of the clusters in Figure 3 are composed of proteins that share the same catalytic type, providing further support that these proteins are homologs. Figure 4 shows the structure similarity network colored by clan. As described in Methods, the clan is the highest level in the MEROPS classification system where proteins are still considered to be evolutionarily related, and the first letter of the clan name represents the catalytic type of a clan's member families.

However, as can be seen in Figure 3A, two of the clusters are of mixed catalytic type. The first cluster includes families C44, T01, T02, and T03 and, consistent with the structure similarity grouping, these have been assigned to one MEROPS clan (clan PB, Figure 4) because they share a common fold and conserved position of the nucleophile, even though the nature of the nucleophile in each family is different [20].

The second mixed cluster (Figure 3A) contains families M14, M17, M20, M28, and C15. Unlike the first cluster discussed above, these families are assigned to different MEROPS clans (Figure 4): MC (M14), MF (M17), MH (M20 and M28), and CF (C15). This is based on differences in catalytic mechanism and non-conserved locations of metal-binding residues [20]. Structural similarity between members of these families has been detected by others and is annotated accordingly in the SCOP structural classification database [59], but opinions differ whether they are evolutionarily related [20], [60]. Strikingly, this is the only cluster in the network that has mixed clans (Figure 4). Viewed at the same level of structure similarity, all other clusters are composed of single clans. In fact, two other unmixed clans (CA and MA) are even more structurally divergent, each emerging as multiple, separate clusters (Figure 4). It is intriguing that the scaffold for this second group of mixed catalytic type shows such variation in catalytic residues and metal-binding positions while sharing similar function. While this question has previously been probed by others, it would be interesting to address this again using the broader context provided by new genomic data.

### Structural analyses of M32 and C51, two families in *T. brucei* that are distant in sequence space from human proteases

One advantage of global views of degradome relationships among species is the ease with which potentially important species differences and similarities can be identified for further investigation. As indicated in Figure 1A, the sequences of two *T. brucei* protease families, M32 and C51, with one and five members, respectively, are quite distant from those of any human proteases. Both M32 and C51 families are known to occur in prokaryotes and parasitic protists [61], [62]. However, as shown in Figure 3A, despite its distance in sequence space from human proteases, the *T. brucei* M32 singleton (TbM32) has several relatively close human structural neighbors. In contrast, the C51 cluster has none.

### The *T. brucei* M32 protease is similar in structure to human ACE and ACE2

In the sequence similarity network, TbM32 (Tb_proteins ID Tb11.02.0100, GI:71754837) fails to show a statistically significant BLAST match to any other protease (the best match has an *E-*value = 0.62). However, as seen in the structure similarity network (Figure 3A), the homology model for TbM32 (“TbM32m”) has several close human structural neighbors: Angiotensin I-converting enzyme (ACE), ACE2, Neurolysin, Thimet oligopeptidase (TOP), and Mitochondrial intermediate peptidase. RMSDs of human crystal structures aligned with TbM32m range from 1.217 Å to 1.283 Å with number of aligned alpha carbon pairs ranging from 73 to 119. TbM32m was created using as a template the crystal structure of an M32 protease from *T. cruzi* (designated here as “TcM32,” PDB code 3DWC). TcM32 is a metallocarboxyeptidase: it cleaves one amino acid from the C-terminus of a peptide and requires a metal ion for activity [61]. Because of its high sequence identity to TcM32 (72%) and the good alignment between predicted active site residues to those of other M32 proteases, we predicted that TbM32 was also likely to be a metallocarboxypeptidase.

**Figure 5:**
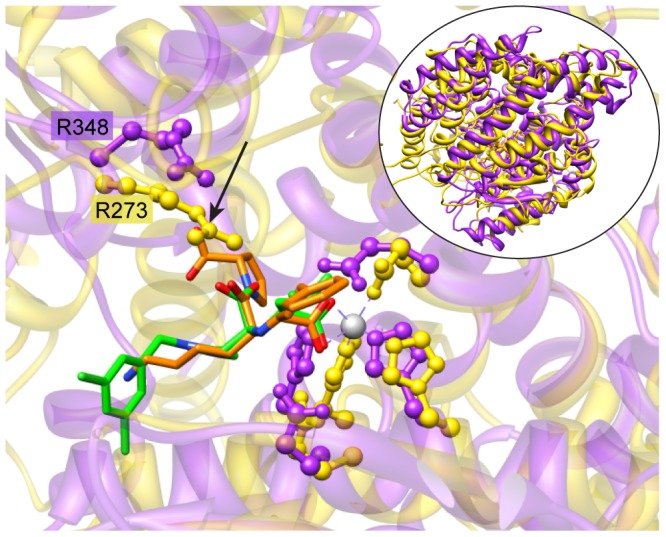
The *T. brucei* M32 protease model shows active site similarity to a human protease ACE2. The model of the *T. brucei* M32 protease (TbM32m, purple) is shown structurally aligned with crystal structure ACE2 (PDB code 1R4L, yellow). Depicted in ball-and-stick representation near the zinc ion are the metal binding residues and catalytic glutamate. ACE2 inhibitor MLN4760 is shown in green and ACE inhibitor lisinopril is in orange stick format (the position of which is from a structural alignment of ACE (1O86) with ACE2). The predicted steric clash of R273 in the ACE2 S1 pocket with lisinopril is marked with an arrow. The R273 CZ of ACE2 is predicted to be 1.5 Å from the lisinopril C9, so that a terminal nitrogen of R273 is in position to overlap with an oxygen of lisinopril. The arginine (R348) from TbM32m that is predicted to be close to the ACE2 R273 is also in ball-and-stick representation. The inset shows the overall structural similarity of the two proteins.

The best characterized human structural neighbor to TbM32m is the anti-hypertensive drug target ACE; however, its lesser known homolog, ACE2, is the only human peptidase in the cluster that is a carboxypeptidase. ACE is a dipeptidyl peptidase, cleaving two residues from the end of a peptide. The presence of an arginine (R273) in the ACE2 S1 binding pocket, instead of the corresponding Gln in ACE, creates a smaller pocket in ACE2 that allows only one residue to fit into the active site C-terminal to the cleavage point. This difference also helps to rationalize the high selectivity of inhibitors for ACE relative to ACE2 [63]. ACE and ACE2 have roles in regulating blood pressure [64] and cardiac function [65], respectively. A number of inhibitors have been designed for ACE [66], and a few have been also developed for ACE2 [45], [67]. Figure 5 (inset) shows the overall structural similarity between TbM32m and ACE2.

We created a structural alignment of TbM32m and the crystal structure for ACE2 bound with inhibitor MLN-4760 (PDB code 1R4L) [63], which showed that a TbM32m arginine (R348) likely corresponds to ACE2 R273 (Figure 5). However R348 is somewhat receded, resulting in more space in the binding pocket in TbM32m. In the TcM32 crystal structure, an arginine superimposes closely with TbM32m R348, resulting in a similar binding pocket space. It is known that ACE inhibitors do not bind to TcM32 ([38] and *personal communication with JJ Cazzulo*) despite an apparently slightly larger pocket than ACE2. Some insight may be given towards understanding this by the knowledge that ACE2 and some other metallopeptidases undergo significant “hinge closure” upon binding a ligand [63]. Model TbM32m was built using apoenzyme TcM32 as a template and it seems feasible that both TbM32 and TcM32 could also undergo hinge closure upon ligand binding, thereby leading to a smaller binding pocket and consequent inability to bind ACE inhibitors. Visual inspection of the structural alignment of TbM32m and ACE2 showed similar binding pocket shapes and no steric clashes of TbM32m residues with the superimposed ACE2 inhibitor, leading to a further prediction that ACE2 inhibitors might bind TbM32.

**Figure 6:**
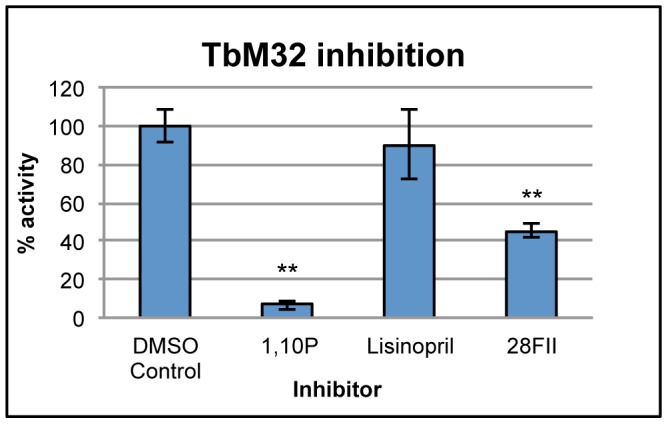
TbM32 is inhibited by 28FII (ACE2 inhibitor) and not by lisinopril (ACE inhibitor). The chart shows results from a representative experiment with 1,10P (1,10 Phenanthroline, 100 µM), lisinopril (10 µM), and 28FII (10 µM). ** indicates significant difference from the control (DMSO vehicle) at p<0.005. The positive control 1,10P is a metal chelator that inhibits metallopeptidases.

To test these two computational predictions, we first cloned and expressed TbM32 and showed that it cleaves the synthetic carboxypeptidase substrate FA-Phe-Phe. Further, it is inhibited by the metal chelator 1,10P (1,10 Phenanthroline). This is consistent with a metallocarboxypeptidase function (Figure 6). We then assayed the recombinant TbM32 with ACE2 inhibitor 28FII, a phosphinic peptide that mimics the transition state structure of ACE2 substrates [45] (MLN-4760 is no longer available). The ACE2 inhibitor produced statistically significant inhibition of TbM32 whereas the ACE inhibitor lisinopril did not (Figure 6). The IC50 of 28FII with human ACE2 has not been published, but its K_i_ is low (0.13 nM) [45], suggesting its potential as an inhibitor. For comparison, the compound MLN-4760 has an IC50 of 0.44 nM with human ACE2 [63]. Our results show that significant inhibition of TbM32 by an ACE2 inhibitor occurs at 10 µM; while this level is higher than the IC50 of an inhibitor designed for ACE2 when used with ACE2, these preliminary results suggest that ACE2 inhibitors may be worth consideration as lead compounds for further development against TbM32.

Although TbM32m and ACE2 are highly similar in overall structure, the α-carbon of the TbM32m R273 originates from a different secondary structure element and has a different topology than ACE2 (Supplementary Figure S1). The sequence identity between these two proteins is <10% so that structural data was needed to give insight into ligand specificity in these highly divergent proteins.

### Predicted structures of the *T. brucei* C51 protease-like sequences differ from those of human proteases

The *T. brucei* C51 family has five members, with pairwise sequence identity among them ranging from 27%–96%. This cluster is remote from human proteases both in terms of sequence and structure. One of the five sequences has been previously identified as *T. brucei* trypanothione synthetase-amidase (“TbTSA”, Tb_proteins ID Tb927.2.4370, GI:84043680) and is the only protein in the cluster that has been experimentally characterized. TbTSA has a C-terminal synthetase domain that catalyzes the production of trypanothione (TSH) from two glutathione (GSH) and one spermidine (Spd) molecule. Its N-terminal amidase (C51) domain catalyzes the reverse reactions [68]. The biological role of the amidase domain is not completely clear, but it likely plays a role in maintaining a concentration balance between these compounds [69]. Unlike TbTSA, the other *T. brucei* C51 sequences have only the amidase domain; a multiple sequence alignment of all five sequences (not shown) indicates that the active site residues predicted to be associated with peptidase/amidase activity are well aligned. We modeled all five amidase domains using as the template the TSA from *L. major* (“LmTSA”, PDB code 2VOB), two of which are represented in Figure 3A (see Methods). The amidase domain of TbTSA (“TbTSAA”) has 58% sequence ID to LmTSA. A structural alignment of each *T. brucei* C51 model to the model of TbTSAA (“TbTSAAm”) (not shown) shows conservation of residues near the active site (an average of 9 of 17 selected pairs were strictly conserved), suggesting that one or more of the uncharacterized C51s may have amidase activity. RMSDs of overall alignments of the *T. brucei* C51 models to TbTSAAm ranged from 0.282–0.586 Å with number of aligned alpha carbon pairs ranging from 95–122.

GSH serves in anti-oxidant and detoxification roles in most animals and plants [70]. However, trypanothione (TSH) serves this purpose in trypanosomes [71] and does not occur in humans. Experiments by others have suggested TbTSA has promise as a drug target [69], [72], [73]. One gene knockout study suggested it is the trypanothione synthetase domain and not the amidase domain that is essential to the parasite; however, this study also showed that both domains are important for parasite virulence [69]. It may be that both domains, perhaps in tandem, are worthy of consideration as drug targets due to their physical connection as a two-domain protein and the biochemical relationship in their roles, i.e., synthesis and hydrolysis of trypanothione. For example, a trypanothione-like inhibitor that can bind both domains may be worth consideration. Additionally, the amidase domain's distinctive structure among prokaryotes and parasitic protozoa, but not in humans [62], makes it an intriguing subject for other cross-species comparisons and for exploring possible drug targets in non-trypanosomes.

**Figure 7:**
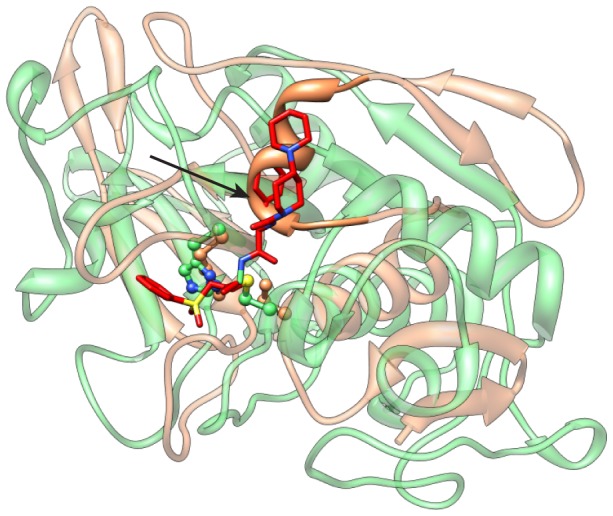
Structure alignment of *T. brucei* C51 model (TbC51m) with a distant structure homolog, human Cathepsin F (CatF). The superposition shows these two proteins have some general, overall structural similarities, but also large differences near the active site. The TbC51 model is colored in light orange, and the human CatF is in light green. While the catalytic Cys-His dyads are closely superimposed (depicted in ball-and-stick), a striking difference is marked by an arrow indicating the predicted steric clash between the CatF vinyl sulfone inhibitor (red) and the helix of TbC51 that partially obstructs the active site.

The closest human structure neighbor to TbTSAAm is cathepsin F (“CatF”, PDB 1M6D) (FAST SN score = 2.6, about 5% sequence ID). Cathepsins are well-studied cysteine proteases in the C01 “papain” family, with roles that range from general protein degradation to wound healing [74]. A number of inhibitors have been developed for this family [49]. The alignment of TbTSAAm with 1M6D, which is complexed with a vinyl sulfone inhibitor (4-morpholin-4-yl-piperidine-1-carboxylic acid [1-(3-benzenesulfonyl-1-propyl-allycarbamoyl)-2-phenylethyl]-amide), shows some general, overall structure similarity (RMSD = 1.1 Å over 16 alpha carbon pairs), but also some striking differences (Figure 7). Most importantly, we predict that a helix exists near the binding site in TbTSAA that could obstruct the binding of a cathepsin-like inhibitor, a feature that is absent in CatF (1M6D).

Upon searching the PDB, the most similar structure to TbTSAAm co-crystallized with a ligand was found to be the amidase domain of *E. coli* glutathionylspermidine (Gsp) synthetase/amidase (“EcGspSAA”, PDB 3A2Y, 36% sequence ID to TbTSAA). Superposition of TbTSAAm with EcGspSAA (RMSD = 0.85 Å over 120 alpha carbon pairs) and human CatF showed that EcGspSAA has a binding site helix similar to the one in TbTSAAm. The TSH-related Gsp binds in a different orientation and location than the protease inhibitor binds human CatF. These observations suggest that such differences in architecture may allow the design of TbTSAA-specific inhibitors that would not cross-react with human C01 peptidases.

## Conclusions

The explosion of data in sequence and structure databases in recent years, along with advances in modeling technology, presents researchers with the opportunity for creating more global views of sequence and structure space from whole organisms than has been possible previously. It has been estimated that sufficient structural templates exist for modeling about 50% of all known proteins [75]. However, leveraging existing data and synthesizing the information into a form that is interpretable in an intuitive and accurate way can be challenging.

We constructed networks presenting the first global views of the degradome landscapes of the parasite, *T. brucei* and its human host, allowing a side-by-side comparison of sequence and structure similarity of predicted active proteases between the two species. The networks show patterns of abundance and variety of proteases while highlighting sequence clusters in which structures are sparse and may be of higher priority for solving new structures. These networks also give clues as to how divergent proteins might be related.

In addition, such global views can give insights about potential drug targets. Our results suggest that ACE2 inhibitors might serve as lead compounds for inhibitor development against TbM32. Also, we predict that uncharacterized C51 members may have an amidase function and that structural differences relative to human peptidases may make it possible to design specific inhibitors for this family of parasite proteins.

Studies such as these should prove more useful as databases of sequence, structure, and function continue to grow and species-specific proteomes become more complete. The networks are available for download and can be viewed and manipulated interactively using the freely available program Cytoscape (www.cytoscape.org).

## Supporting Information

Figure S1 **Alignment of** ***T. brucei*** **M32 (TbM32) and human ACE2 shows great differences in sequence and topology.** The structure-based sequence alignment shown here illustrates that, despite having similar overall structure and active site architectures, these proteins are distant from each other by sequence, and functionally important corresponding arginines that are located in similar positions in 3D space have different origins in topological space in the two proteins. Secondary structure is shown as: alpha helix = squiggles; beta strand = arrow; turn = T. Highlighted in yellow and with arrows are the arginines from TbM32 (R348) and ACE2 (R273) in the S1 pocket that are likely critical for inhibitor specificity and protein function determination.
(TIF)

Table S1 **Predicted active peptidases, counts by family and clan.** (A) Counts are by descending order of peptidases predicted to be active in humans and *T. brucei* according to assigned MEROPS family (see Methods). * indicates a family is not found in the other species. (B) Counts by assigned MEROPS clan (see Methods) show that the three largest clans in humans are also prominent in *T. brucei* except for clan PA, which includes the trypsin/chymotrypsin (S01) family, which is devoid of clan members in *T. brucei.*
(DOCX)

Table S2 **Human and** ***T. brucei*** **sequence-similar clusters with no structure representation.** Human and *T. brucei* clusters of size ≥3 are shown if they have no structure representation (no crystal structures and no homology models). There may be other structure-represented clusters in the sequence-similarity network with members of the same families as shown here, but the clusters here are composed of sequences divergent from any other cluster (*E-*value >1e^−5^). Family A22 has two sequence-divergent clusters in the network with no structure representation.
(DOCX)
