# Supplementary figures and images for: A Global Comparison of the Human and T. brucei Degradomes Gives Insights about Possible Parasite Drug Targets
Source: PLoS Negl Trop Dis. 2012 Dec 6;6(12):e1942. doi: 10.1371/journal.pntd.0001942 (PMC3516576; doi:10.1371/journal.pntd.0001942)

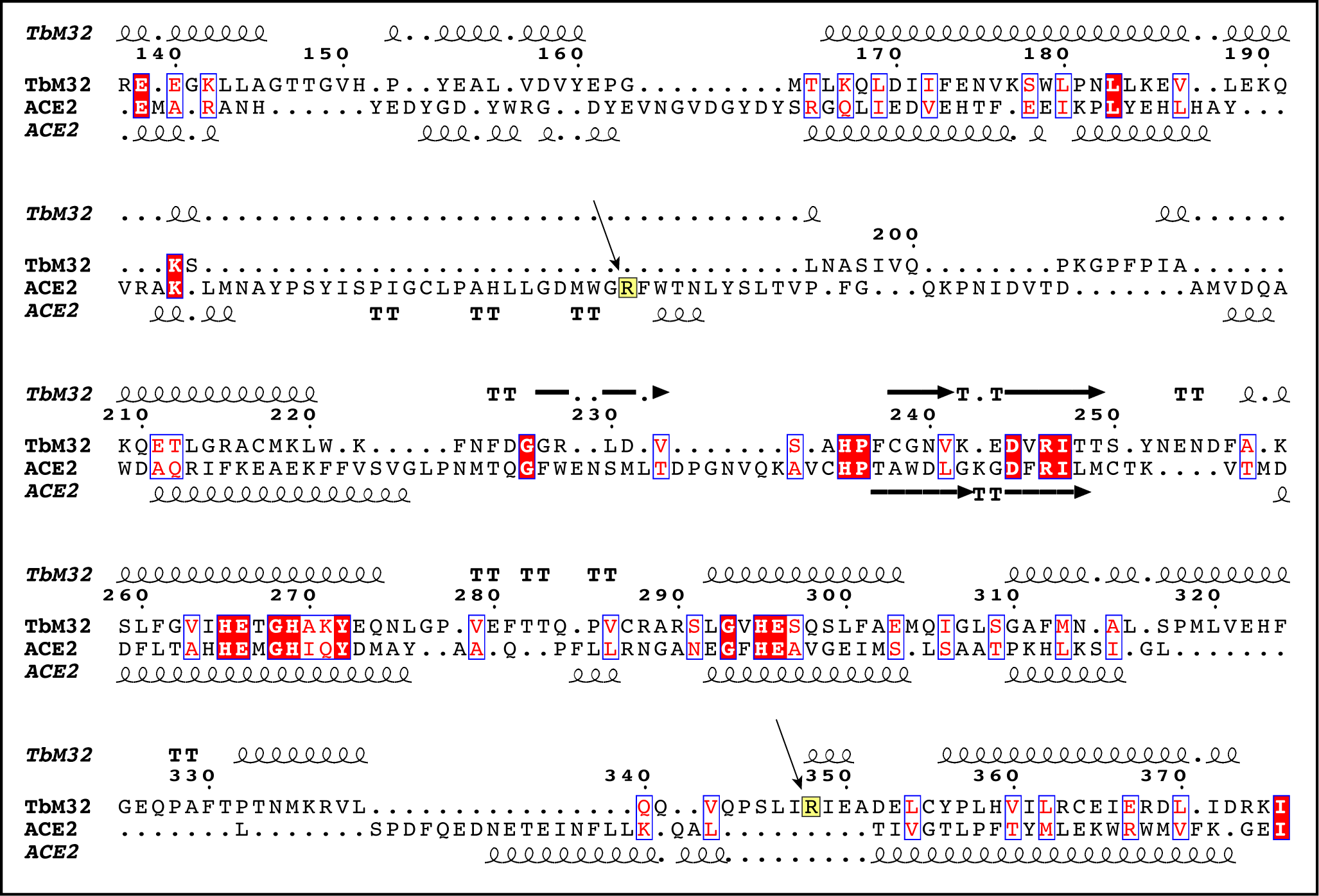

Supplement: Figure S1 — Alignment of T. brucei M32 (TbM32) and human ACE2 shows great differences in sequence and topology. The structure-based sequence alignment shown here illustrates that, despite having similar overall structure and active site architectures, these proteins are distant from each other by sequence, and functionally important corresponding arginines that are located in similar positions in 3D space have different origins in topological space in the two proteins. Secondary structure is shown as: alpha helix = squiggles; beta strand = arrow; turn = T. Highlighted in yellow and with arrows are the arginines from TbM32 (R348) and ACE2 (R273) in the S1 pocket that are likely critical for inhibitor specificity and protein function determination. (TIF) [file pntd.0001942.s001.tif]
