# Supplementary material for: A Global Comparison of the Human and T. brucei Degradomes Gives Insights about Possible Parasite Drug Targets
Source: PLoS Negl Trop Dis. 2012 Dec 6;6(12):e1942. doi: 10.1371/journal.pntd.0001942 (PMC3516576; doi:10.1371/journal.pntd.0001942)
Supplement: Table S1 — Predicted active peptidases, counts by family and clan. (A) Counts are by descending order of peptidases predicted to be active in humans and T. brucei according to assigned MEROPS family (see Methods). * indicates a family is not found in the other species. (B) Counts by assigned MEROPS clan (see Methods) show that the three largest clans in humans are also prominent in T. brucei except for clan PA, which includes the trypsin/chymotrypsin (S01) family, which is devoid of clan members in T. brucei. (DOCX) [file pntd.0001942.s002.docx]

**Table S1A. Predicted active peptidases, counts by family.** Counts are by descending order of peptidases predicted to be active in humans and *T. brucei* according to assigned MEROPS family (see Methods). * indicates a family is not found in the other species.

| **Human** | | ***T. brucei*** | |
| --- | --- | --- | --- |
| **family** | **count** | **family** | **count** |
| S01* | 115 | C01 | 12 |
| C19 | 51 | C19 | 12 |
| A02* | 46 | M08 | 12 |
| M12* | 40 | S09 | 10 |
| S09 | 26 | T01 | 7 |
| M10* | 22 | C51* | 5 |
| M14* | 15 | M20 | 5 |
| C14 | 14 | M24 | 5 |
| M01 | 14 | M41 | 5 |
| S63* | 14 | S33 | 5 |
| C02 | 13 | C14 | 4 |
| T01 | 13 | M01 | 4 |
| C01 | 12 | M67 | 4 |
| A01* | 11 | M16 | 3 |
| S08 | 10 | M17 | 3 |
| S33 | 10 | S10 | 3 |
| C48 | 8 | A22 | 2 |
| T03* | 8 | C12 | 2 |
| A22 | 7 | C54 | 2 |
| M13* | 7 | C85 | 2 |
| M24 | 7 | M03 | 2 |
| M28* | 7 | S08 | 2 |
| M67 | 7 | S26 | 2 |
| C85 | 6 | C02 | 1 |
| C64* | 5 | C13 | 1 |
| M41 | 5 | C15 | 1 |
| S26 | 5 | C26 | 1 |
| S54* | 5 | C48 | 1 |
| C12 | 4 | C50 | 1 |
| C54 | 4 | C65 | 1 |
| C86* | 4 | C78 | 1 |
| M16 | 4 | C88 | 1 |
| M20 | 4 | M18 | 1 |
| C13 | 3 | M22 | 1 |
| C26 | 3 | M32* | 1 |
| C44* | 3 | M48 | 1 |
| C46* | 3 | M76 | 1 |
| C69* | 3 |  |  |
| M02* | 3 |  |  |
| M03 | 3 |  |  |
| M17 | 3 |  |  |
| S10 | 3 |  |  |
| S28* | 3 |  |  |
| T02* | 3 |  |  |
| C15 | 2 |  |  |
| C65 | 2 |  |  |
| C78 | 2 |  |  |
| M19* | 2 |  |  |
| M22 | 2 |  |  |
| M43* | 2 |  |  |
| M48 | 2 |  |  |
| M54* | 2 |  |  |
| S16* | 2 |  |  |
| S68* | 2 |  |  |
| T06* | 2 |  |  |
| C50 | 1 |  |  |
| C67* | 1 |  |  |
| C88 | 1 |  |  |
| C89* | 1 |  |  |
| M08 | 1 |  |  |
| M18 | 1 |  |  |
| M49* | 1 |  |  |
| M50* | 1 |  |  |
| M76 | 1 |  |  |
| S12* | 1 |  |  |
| S14* | 1 |  |  |
| S53* | 1 |  |  |
| S59* | 1 |  |  |
| S60* | 1 |  |  |
| S71* | 1 |  |  |
| S72* | 1 |  |  |

**Table S1B.** **Predicted active peptidases, counts by family and clan.** Counts by assigned MEROPS clan (see Methods) show that the three largest clans in humans are also prominent in *T. brucei* except for clan PA, which includes the trypsin/chymotrypsin (S01) family, which is devoid of clan members in *T. brucei.*

| **clan** | **human** | ***T. brucei*** |
| --- | --- | --- |
| AA | 57 | 0 |
| AD | 7 | 2 |
| CA | 105 | 39 |
| CD | 18 | 6 |
| CE | 8 | 1 |
| CF | 2 | 1 |
| CH | 3 | 0 |
| M- | 2 | 1 |
| MA | 101 | 25 |
| MC | 15 | 0 |
| ME | 4 | 3 |
| MF | 3 | 3 |
| MG | 7 | 5 |
| MH | 12 | 6 |
| MJ | 2 | 0 |
| MK | 2 | 1 |
| MM | 1 | 0 |
| MP | 7 | 4 |
| PA | 115 | 0 |
| PB | 33 | 7 |
| PC | 3 | 1 |
| S- | 18 | 0 |
| SB | 11 | 2 |
| SC | 42 | 18 |
| SE | 1 | 0 |
| SF | 5 | 2 |
| SJ | 2 | 0 |
| SK | 1 | 0 |
| SP | 1 | 0 |
| SR | 1 | 0 |
| ST | 5 | 0 |
| TOTAL | 594 | 127 |
