# Supplementary material for: A Global Comparison of the Human and T. brucei Degradomes Gives Insights about Possible Parasite Drug Targets
Source: PLoS Negl Trop Dis. 2012 Dec 6;6(12):e1942. doi: 10.1371/journal.pntd.0001942 (PMC3516576; doi:10.1371/journal.pntd.0001942)
Supplement: Table S2 — Human and T. brucei sequence-similar clusters with no structure representation. Human and T. brucei clusters of size ≥3 are shown if they have no structure representation (no crystal structures and no homology models). There may be other structure-represented clusters in the sequence-similarity network with members of the same families as shown here, but the clusters here are composed of sequences divergent from any other cluster (E-value >1e−5). Family A22 has two sequence-divergent clusters in the network with no structure representation. (DOCX) [file pntd.0001942.s003.docx]

**Table S2.** **Human and *T. brucei* sequence-similar clusters with no structure representation.** Human and *T. brucei* clusters of size ≥ 3 are shown if they have no structure representation (no crystal structures and no homology models). There may be other structure-represented clusters in the sequence-similarity network with members of the same families as shown here, but the clusters here are composed of sequences divergent from any other cluster (*E-*value > 1e^-5^). Family A22 has two sequence-divergent clusters in the network with no structure representation.

| **family** | ***H. sapiens*** | ***T. brucei*** | **total cluster size** |
| --- | --- | --- | --- |
| A02 | 38 | 0 | 38 |
| M08 | 1 | 12 | 13 |
| C85 | 6 | 2 | 8 |
| A02 | 8 | 0 | 8 |
| A22 | 5 | 1 | 6 |
| C14 | 0 | 4 | 4 |
| C13 | 3 | 1 | 4 |
| A22 | 2 | 1 | 3 |
